# 2-(2-{2-[2-(Dibromo­meth­yl)phen­oxy]eth­oxy}benz­yloxy)benzaldehyde

**DOI:** 10.1107/S160053681100643X

**Published:** 2011-02-26

**Authors:** Juan Xia, Xiang Liu, An-Qi Wang, Zhong-Xing Su

**Affiliations:** a State Key Laboratory of Applied Organic Chemistry, College of Chemistry and Chemical Engineering, Lanzhou University, Lanzhou 730000, People’s Republic of China

## Abstract

The mol­ecule of the title compound, C_23_H_20_Br_2_O_4_, adopts a *Z* conformation as a result of inter­molecular C—H⋯Br bonding. One benzene ring, with the structure *R*-CHBr_2_, makes a dihedral angle of 63.0 (2)° with the other benzene ring attached to the aldehyde group. Inter­molecular π–π stacking inter­actions [centroid–centroid distance = 3.698 (4) Å] and a weak C—H⋯Br contact is present in the crystal structure.

## Related literature

For general background to the biological activity of salicyl­aldehydes and their derivatives, see: Jahnke *et al.* (1993 ▶); Pelttari *et al.* (2007 ▶); Fillebeen & Pantopoulos (2010 ▶); Fan *et al.* (2010 ▶). For related structures, see: Mori *et al.* (2010 ▶); Potapov *et al.* (2009 ▶); Purushothaman & Raghunathan (2009 ▶). For the preparation of the title compound, see: Purushothaman & Raghunathan (2009 ▶); Zhang *et al.* (2010 ▶).
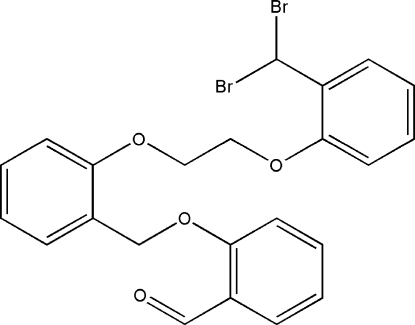


## Experimental

### 

#### Crystal data

C_23_H_20_Br_2_O_4_
*M* *_r_* = 520.19
Monoclinic, 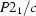

*a* = 12.867 (7) Å
*b* = 18.07 (1) Å
*c* = 9.649 (5) Å
β = 108.955 (6)°
*V* = 2122 (2) Å^3^
*Z* = 4
Mo *K*α radiation
μ = 3.85 mm^−1^
*T* = 296 K
0.34 × 0.32 × 0.28 mm

#### Data collection

Bruker APEXII CCD diffractometer
Absorption correction: multi-scan (*SADABS*; Sheldrick, 1996 ▶) *T*_min_ = 0.281, *T*_max_ = 0.341
15392 measured reflections
3944 independent reflections
1905 reflections with *I* > 2σ(*I*)
*R*_int_ = 0.063

#### Refinement

*R*[*F* ^2^ > 2σ(*F* ^2^)] = 0.062
*wR*(*F* ^2^) = 0.207
*S* = 1.02
3944 reflections
263 parameters
H-atom parameters constrained
Δρ_max_ = 0.79 e Å^−3^
Δρ_min_ = −0.75 e Å^−3^

### 

Data collection: *APEX2* (Bruker, 2007 ▶); cell refinement: *SAINT* (Bruker, 2007 ▶); data reduction: *SAINT*; program(s) used to solve structure: *SHELXS97* (Sheldrick, 2008 ▶); program(s) used to refine structure: *SHELXL97* (Sheldrick, 2008 ▶); molecular graphics: *SHELXTL* (Sheldrick, 2008 ▶); software used to prepare material for publication: *SHELXTL*.

## Supplementary Material

Crystal structure: contains datablocks I, global. DOI: 10.1107/S160053681100643X/rk2257sup1.cif

Structure factors: contains datablocks I. DOI: 10.1107/S160053681100643X/rk2257Isup2.hkl

Additional supplementary materials:  crystallographic information; 3D view; checkCIF report

## Figures and Tables

**Table 1:** Hydrogen-bond geometry (Å, °)

*D*—H⋯*A*	*D*—H	H⋯*A*	*D*⋯*A*	*D*—H⋯*A*
C5—H5⋯Br1^i^	0.93	3.03	3.529 (7)	116

Symmetry code: (i) 
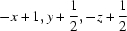
.

**Fig. 1.**
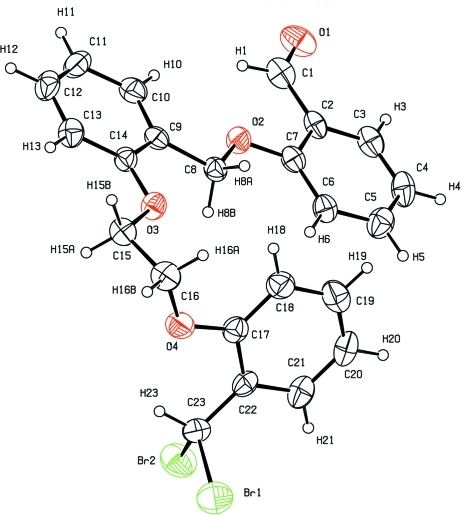
The molecular structure of the title compound with the atom numbering scheme. Displacement ellipsoids are drawn at the 30% probability level. H atoms are presented as a small spheres of arbitrary radius.

**Fig. 2.**
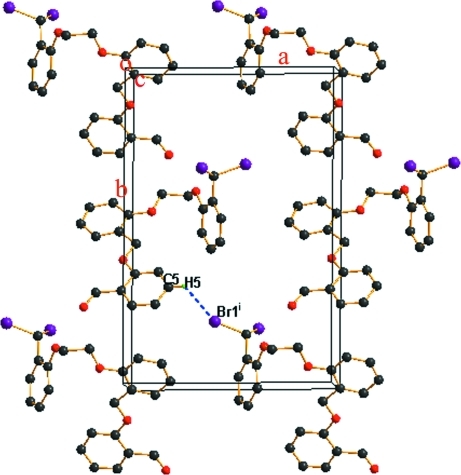
A view of the C—H···Bri interactions in the crystal structure of the title compound. Symmetry code: (i) -x+1, y+1/2, -z+1/2).

## supplementary crystallographic information

### Comment

It is reported that salicylaldehydes and their derivatives have showed a wide variety of biological activities, such as antiseptic, labelling cell, antiproliferative and pesticidal (Jahnke *et al.*, 1993; Pelttari *et al.*, 2007; Fillebeen & Pantopoulos, 2010; Fan *et al.*, 2010). As an important class of aldehydes, substituted aldehydes also exhibit potential biological activities. The related structures  also have been reported (Mori *et al.*, 2010; Potapov *et al.*, 2009; Purushothaman & Raghunathan, 2009). On this base, the title compound was synthesized.

In the title compound (Fig. 1), a dihedral angle 63.0 (2)° is observed between benzene rings on the both ends of molecule. The crystal structure is stabilized by weak intramolecular C—H···O bonds.

The molecule of the title compound is linked by the C—H···Br bonding (Fig. 2) in to the *Z* formation.

Furthermore, the weak intermolecular π–π stacking interactions - *Cg*1···*Cg*2^ii^= 3.698 (4)Å, *Cg*3···*Cg*3^iii^ = 4.193 (5)Å, where *Cg*1 is centroid of the ring C2–C7, *Cg*2 is centroid of the ring C9–C14 and *Cg*3 is centroid of the ring C17–C22. Symmetry codes: (ii) -*x*, 1-*y*, -*z*; (iii) 1-*x*, 1-*y*, 1-*z*.

### Experimental

All reagents and solvents were obtained from commercial sources and needed to be further purified. The title compound was synthesized according to the related literature (Purushothaman & Raghunathan, 2009). A solution of salicylaldehyde (2 mmol in 10 ml acetone) was slowly added dropwise to a suspension of 1,2-bis(2-(bromomethyl)phenoxy) ethane (1 mmol in 20 ml acetone) prepared according to the reported method (Zhang *et al.*, 2010) and anhydrous potassium carbonate (2 mmol). The mixture was refluxed for 8 h. The reaction mixture was then cooled to room temperature and filtered. After this period, the residue was dissolved and extracted by ethyl acetate. The combined organical layer was washed with water and then dried with anhydrous sodium sulfate. After that the solvent was evaporated under vacuum to give the product. The obtained residue was purified by flash column chromatography on silica gel using petroleum ether/ethylacetate (5:2) mixtures as eluent.

### Refinement

All H atoms were found from difference Fourier maps and were subsequently refined in a riding-model approximation with C—H distances ranging from 0.93Å to 0.98Å and with *U*_iso_(H) = 1.2 *U*_eq_(C) of the carrier atom.

### Crystal data

**Table:**

C_23_H_20_Br_2_O_4_	*F*(000) = 1040
*M**_r_* = 520.19	*D*_x_ = 1.628 Mg m^−^^3^
Monoclinic, *P*2_1_/*c*	Mo *K*α radiation, λ = 0.71073 Å
Hall symbol: -P 2ybc	Cell parameters from 1808 reflections
*a* = 12.867 (7) Å	θ = 2.3–17.5°
*b* = 18.07 (1) Å	µ = 3.85 mm^−^^1^
*c* = 9.649 (5) Å	*T* = 296 K
β = 108.955 (6)°	Block, colourless
*V* = 2122 (2) Å^3^	0.34 × 0.32 × 0.28 mm
*Z* = 4	

### Data collection

**Table:**

Bruker APEXII CCD diffractometer	3944 independent reflections
Radiation source: fine-focus sealed tube	1905 reflections with *I* > 2σ(*I*)
graphite	*R*_int_ = 0.063
φ and ω scans	θ_max_ = 25.5°, θ_min_ = 2.3°
Absorption correction: multi-scan (*SADABS*; Sheldrick, 1996)	*h* = −15→15
*T*_min_ = 0.281, *T*_max_ = 0.341	*k* = −21→21
15392 measured reflections	*l* = −11→11

### Refinement

**Table:**

Refinement on *F*^2^	Secondary atom site location: difference Fourier map
Least-squares matrix: full	Hydrogen site location: inferred from neighbouring sites
*R*[*F*^2^ > 2σ(*F*^2^)] = 0.062	H-atom parameters constrained
*wR*(*F*^2^) = 0.207	*w* = 1/[σ^2^(*F*_o_^2^) + (0.1049*P*)^2^ + 0.4797*P*] where *P* = (*F*_o_^2^ + 2*F*_c_^2^)/3
*S* = 1.02	(Δ/σ)_max_ < 0.001
3944 reflections	Δρ_max_ = 0.79 e Å^−^^3^
263 parameters	Δρ_min_ = −0.75 e Å^−^^3^
0 restraints	Extinction correction: *SHELXL97* (Sheldrick, 2008), Fc^*^=kFc[1+0.001xFc^2^λ^3^/sin(2θ)]^-1/4^
Primary atom site location: structure-invariant direct methods	Extinction coefficient: 0.0020 (3)

### Special details

**Table:**

Geometry. All s.u.'s (except the s.u. in the dihedral angle between two l.s. planes) are estimated using the full covariance matrix. The cell s.u.'s are taken into account individually in the estimation of s.u.'s in distances, angles and torsion angles; correlations between s.u.'s in cell parameters are only used when they are defined by crystal symmetry. An approximate (isotropic) treatment of cell s.u.'s is used for estimating s.u.'s involving l.s. planes.
Refinement. Refinement of *F*^2^ against ALL reflections. The weighted *R*-factor *wR* and goodness of fit *S* are based on *F*^2^, conventional *R*-factors *R* are based on *F*, with *F* set to zero for negative *F*^2^. The threshold expression of *F*^2^ > σ(*F*^2^) is used only for calculating *R*-factors(gt) *etc*. and is not relevant to the choice of reflections for refinement. *R*-factors based on *F*^2^ are statistically about twice as large as those based on *F*, and *R*-factors based on ALL data will be even larger.

### Fractional atomic coordinates and isotropic or equivalent isotropic displacement parameters (Å^2^)

**Table:**

	*x*	*y*	*z*	*U*_iso_*/*U*_eq_	
Br1	0.56817 (7)	0.30130 (6)	0.29797 (13)	0.1092 (5)	
Br2	0.37069 (9)	0.31794 (5)	−0.00106 (10)	0.0986 (5)	
C1	−0.1269 (7)	0.7137 (4)	−0.1294 (8)	0.069 (2)	
H1	−0.1621	0.6777	−0.0919	0.083*	
C2	−0.0059 (6)	0.7075 (3)	−0.0932 (6)	0.0509 (16)	
C3	0.0495 (7)	0.7556 (4)	−0.1572 (7)	0.0658 (19)	
H3	0.0096	0.7922	−0.2199	0.079*	
C4	0.1583 (7)	0.7514 (4)	−0.1323 (8)	0.073 (2)	
H4	0.1932	0.7846	−0.1761	0.088*	
C5	0.2176 (6)	0.6962 (4)	−0.0397 (8)	0.070 (2)	
H5	0.2930	0.6929	−0.0213	0.084*	
C6	0.1660 (6)	0.6458 (4)	0.0260 (7)	0.0551 (16)	
H6	0.2062	0.6085	0.0865	0.066*	
C7	0.0548 (6)	0.6522 (3)	−0.0001 (6)	0.0511 (16)	
C8	0.0522 (5)	0.5531 (3)	0.1655 (7)	0.0487 (15)	
H8A	0.1057	0.5775	0.2474	0.058*	
H8B	0.0905	0.5182	0.1229	0.058*	
C9	−0.0317 (5)	0.5130 (3)	0.2169 (6)	0.0470 (15)	
C10	−0.1419 (6)	0.5287 (4)	0.1643 (7)	0.0617 (18)	
H10	−0.1676	0.5664	0.0961	0.074*	
C11	−0.2162 (6)	0.4878 (5)	0.2135 (8)	0.070 (2)	
H11	−0.2910	0.4979	0.1766	0.084*	
C12	−0.1791 (7)	0.4340 (4)	0.3138 (8)	0.071 (2)	
H12	−0.2284	0.4073	0.3465	0.085*	
C13	−0.0685 (6)	0.4184 (4)	0.3684 (7)	0.0602 (17)	
H13	−0.0436	0.3811	0.4378	0.072*	
C14	0.0057 (5)	0.4576 (3)	0.3211 (7)	0.0487 (16)	
C15	0.1611 (5)	0.3890 (3)	0.4722 (6)	0.0536 (16)	
H15A	0.1381	0.3412	0.4268	0.064*	
H15B	0.1347	0.3939	0.5552	0.064*	
C16	0.2849 (6)	0.3950 (4)	0.5223 (7)	0.0670 (19)	
H16A	0.3066	0.4437	0.5639	0.080*	
H16B	0.3163	0.3586	0.5984	0.080*	
C17	0.3652 (5)	0.4407 (4)	0.3435 (7)	0.0556 (17)	
C18	0.3584 (6)	0.5154 (4)	0.3772 (8)	0.0675 (19)	
H18	0.3226	0.5295	0.4426	0.081*	
C19	0.4055 (6)	0.5681 (4)	0.3121 (9)	0.077 (2)	
H19	0.4046	0.6176	0.3381	0.092*	
C20	0.4531 (6)	0.5485 (5)	0.2106 (9)	0.076 (2)	
H20	0.4825	0.5846	0.1656	0.091*	
C21	0.4579 (6)	0.4750 (5)	0.1743 (8)	0.071 (2)	
H21	0.4906	0.4622	0.1047	0.086*	
C22	0.4145 (5)	0.4197 (4)	0.2401 (7)	0.0536 (16)	
C23	0.4210 (6)	0.3400 (4)	0.2060 (8)	0.0657 (19)	
H23	0.3725	0.3135	0.2490	0.079*	
O1	−0.1819 (4)	0.7608 (3)	−0.2023 (5)	0.0824 (16)	
O2	−0.0053 (3)	0.6067 (2)	0.0578 (4)	0.0549 (11)	
O3	0.1171 (4)	0.4474 (2)	0.3681 (4)	0.0560 (11)	
O4	0.3266 (4)	0.3834 (2)	0.4054 (5)	0.0737 (14)	

### Atomic displacement parameters (Å^2^)

**Table:**

	*U*^11^	*U*^22^	*U*^33^	*U*^12^	*U*^13^	*U*^23^
Br1	0.0693 (7)	0.0994 (8)	0.1485 (10)	0.0213 (5)	0.0212 (6)	0.0112 (6)
Br2	0.1243 (9)	0.0954 (7)	0.0791 (7)	0.0014 (5)	0.0372 (6)	−0.0179 (5)
C1	0.084 (6)	0.059 (4)	0.059 (5)	0.000 (4)	0.015 (4)	0.000 (4)
C2	0.070 (5)	0.037 (3)	0.042 (4)	−0.006 (3)	0.013 (3)	−0.004 (3)
C3	0.087 (6)	0.052 (4)	0.054 (4)	−0.005 (4)	0.017 (4)	−0.002 (3)
C4	0.096 (6)	0.059 (5)	0.065 (5)	−0.022 (4)	0.027 (5)	0.010 (4)
C5	0.063 (5)	0.082 (5)	0.064 (5)	−0.015 (4)	0.019 (4)	−0.008 (4)
C6	0.058 (5)	0.051 (4)	0.049 (4)	−0.005 (3)	0.008 (3)	−0.001 (3)
C7	0.062 (5)	0.046 (4)	0.041 (4)	−0.004 (3)	0.011 (3)	−0.007 (3)
C8	0.056 (4)	0.040 (3)	0.051 (4)	0.001 (3)	0.019 (3)	0.004 (3)
C9	0.056 (4)	0.044 (4)	0.042 (4)	0.000 (3)	0.016 (3)	−0.010 (3)
C10	0.073 (5)	0.055 (4)	0.060 (4)	0.005 (4)	0.026 (4)	−0.005 (3)
C11	0.051 (4)	0.087 (5)	0.077 (5)	−0.013 (4)	0.030 (4)	−0.016 (5)
C12	0.083 (6)	0.078 (5)	0.071 (5)	−0.028 (4)	0.049 (5)	−0.019 (4)
C13	0.069 (5)	0.062 (4)	0.051 (4)	−0.008 (4)	0.021 (4)	0.002 (3)
C14	0.058 (5)	0.045 (4)	0.049 (4)	−0.005 (3)	0.025 (3)	−0.001 (3)
C15	0.071 (5)	0.056 (4)	0.034 (3)	0.001 (3)	0.017 (3)	0.006 (3)
C16	0.082 (5)	0.069 (5)	0.053 (4)	0.004 (4)	0.025 (4)	0.006 (4)
C17	0.047 (4)	0.060 (4)	0.056 (4)	0.000 (3)	0.012 (3)	0.003 (3)
C18	0.064 (5)	0.065 (5)	0.075 (5)	0.008 (4)	0.024 (4)	0.011 (4)
C19	0.064 (5)	0.057 (4)	0.086 (6)	0.004 (4)	−0.006 (5)	0.003 (4)
C20	0.063 (5)	0.082 (6)	0.079 (6)	−0.023 (4)	0.020 (4)	0.013 (4)
C21	0.061 (5)	0.088 (6)	0.064 (5)	−0.019 (4)	0.018 (4)	−0.002 (4)
C22	0.038 (4)	0.069 (4)	0.052 (4)	−0.005 (3)	0.011 (3)	−0.003 (3)
C23	0.061 (5)	0.064 (4)	0.081 (5)	0.007 (3)	0.035 (4)	0.001 (4)
O1	0.089 (4)	0.072 (3)	0.068 (3)	0.029 (3)	0.000 (3)	0.008 (3)
O2	0.058 (3)	0.050 (3)	0.057 (3)	0.002 (2)	0.019 (2)	0.014 (2)
O3	0.061 (3)	0.058 (3)	0.051 (3)	−0.002 (2)	0.021 (2)	0.011 (2)
O4	0.097 (4)	0.059 (3)	0.084 (3)	0.006 (3)	0.056 (3)	0.007 (3)

### Geometric parameters (Å, °)

**Table:**

Br1—C23	1.941 (7)	C12—C13	1.377 (10)
Br2—C23	1.931 (7)	C12—H12	0.9300
C1—O1	1.182 (8)	C13—C14	1.380 (8)
C1—C2	1.485 (10)	C13—H13	0.9300
C1—H1	0.9300	C14—O3	1.368 (7)
C2—C3	1.389 (9)	C15—O3	1.441 (7)
C2—C7	1.400 (8)	C15—C16	1.510 (9)
C3—C4	1.344 (10)	C15—H15A	0.9700
C3—H3	0.9300	C15—H15B	0.9700
C4—C5	1.389 (10)	C16—O4	1.414 (7)
C4—H4	0.9300	C16—H16A	0.9700
C5—C6	1.395 (9)	C16—H16B	0.9700
C5—H5	0.9300	C17—O4	1.367 (7)
C6—C7	1.376 (9)	C17—C18	1.397 (9)
C6—H6	0.9300	C17—C22	1.397 (9)
C7—O2	1.366 (7)	C18—C19	1.385 (10)
C8—O2	1.437 (7)	C18—H18	0.9300
C8—C9	1.510 (8)	C19—C20	1.361 (11)
C8—H8A	0.9700	C19—H19	0.9300
C8—H8B	0.9700	C20—C21	1.379 (11)
C9—C10	1.372 (9)	C20—H20	0.9300
C9—C14	1.390 (8)	C21—C22	1.394 (9)
C10—C11	1.408 (9)	C21—H21	0.9300
C10—H10	0.9300	C22—C23	1.486 (9)
C11—C12	1.344 (10)	C23—H23	0.9800
C11—H11	0.9300		
			
O1—C1—C2	124.9 (7)	O3—C14—C13	125.8 (6)
O1—C1—H1	117.5	O3—C14—C9	114.6 (5)
C2—C1—H1	117.5	C13—C14—C9	119.7 (6)
C3—C2—C7	118.2 (7)	O3—C15—C16	107.8 (5)
C3—C2—C1	119.9 (6)	O3—C15—H15A	110.1
C7—C2—C1	121.9 (6)	C16—C15—H15A	110.1
C4—C3—C2	122.7 (7)	O3—C15—H15B	110.1
C4—C3—H3	118.7	C16—C15—H15B	110.1
C2—C3—H3	118.7	H15A—C15—H15B	108.5
C3—C4—C5	118.6 (7)	O4—C16—C15	111.6 (5)
C3—C4—H4	120.7	O4—C16—H16A	109.3
C5—C4—H4	120.7	C15—C16—H16A	109.3
C4—C5—C6	121.2 (7)	O4—C16—H16B	109.3
C4—C5—H5	119.4	C15—C16—H16B	109.3
C6—C5—H5	119.4	H16A—C16—H16B	108.0
C7—C6—C5	118.8 (6)	O4—C17—C18	124.7 (6)
C7—C6—H6	120.6	O4—C17—C22	114.9 (6)
C5—C6—H6	120.6	C18—C17—C22	120.4 (6)
O2—C7—C6	124.6 (6)	C19—C18—C17	119.3 (7)
O2—C7—C2	114.9 (6)	C19—C18—H18	120.4
C6—C7—C2	120.5 (6)	C17—C18—H18	120.4
O2—C8—C9	107.8 (5)	C20—C19—C18	120.8 (7)
O2—C8—H8A	110.1	C20—C19—H19	119.6
C9—C8—H8A	110.1	C18—C19—H19	119.6
O2—C8—H8B	110.1	C19—C20—C21	120.1 (7)
C9—C8—H8B	110.1	C19—C20—H20	119.9
H8A—C8—H8B	108.5	C21—C20—H20	119.9
C10—C9—C14	119.3 (6)	C20—C21—C22	121.1 (7)
C10—C9—C8	122.8 (6)	C20—C21—H21	119.5
C14—C9—C8	117.8 (5)	C22—C21—H21	119.5
C9—C10—C11	120.0 (7)	C17—C22—C21	118.2 (7)
C9—C10—H10	120.0	C17—C22—C23	119.5 (6)
C11—C10—H10	120.0	C21—C22—C23	122.3 (6)
C12—C11—C10	120.1 (7)	C22—C23—Br2	113.9 (5)
C12—C11—H11	120.0	C22—C23—Br1	111.4 (5)
C10—C11—H11	120.0	Br2—C23—Br1	110.4 (3)
C11—C12—C13	120.3 (6)	C22—C23—H23	106.9
C11—C12—H12	119.8	Br2—C23—H23	106.9
C13—C12—H12	119.8	Br1—C23—H23	106.9
C14—C13—C12	120.6 (7)	C7—O2—C8	118.4 (5)
C14—C13—H13	119.7	C14—O3—C15	117.5 (5)
C12—C13—H13	119.7	C17—O4—C16	121.6 (5)
			
O1—C1—C2—C3	−6.0 (10)	O3—C15—C16—O4	−63.5 (7)
O1—C1—C2—C7	176.8 (6)	O4—C17—C18—C19	176.8 (6)
C7—C2—C3—C4	−0.6 (10)	C22—C17—C18—C19	−3.0 (10)
C1—C2—C3—C4	−178.0 (6)	C17—C18—C19—C20	3.4 (11)
C2—C3—C4—C5	0.6 (10)	C18—C19—C20—C21	−1.9 (11)
C3—C4—C5—C6	0.3 (10)	C19—C20—C21—C22	0.0 (11)
C4—C5—C6—C7	−1.1 (10)	O4—C17—C22—C21	−178.7 (6)
C5—C6—C7—O2	−179.8 (5)	C18—C17—C22—C21	1.1 (9)
C5—C6—C7—C2	1.1 (9)	O4—C17—C22—C23	0.1 (9)
C3—C2—C7—O2	−179.5 (5)	C18—C17—C22—C23	179.9 (6)
C1—C2—C7—O2	−2.2 (8)	C20—C21—C22—C17	0.4 (10)
C3—C2—C7—C6	−0.3 (9)	C20—C21—C22—C23	−178.3 (7)
C1—C2—C7—C6	177.0 (6)	C17—C22—C23—Br2	130.6 (5)
O2—C8—C9—C10	0.0 (8)	C21—C22—C23—Br2	−50.7 (8)
O2—C8—C9—C14	179.3 (5)	C17—C22—C23—Br1	−103.8 (6)
C14—C9—C10—C11	−1.3 (9)	C21—C22—C23—Br1	75.0 (7)
C8—C9—C10—C11	178.0 (5)	C6—C7—O2—C8	6.8 (8)
C9—C10—C11—C12	1.0 (10)	C2—C7—O2—C8	−174.1 (5)
C10—C11—C12—C13	−0.4 (10)	C9—C8—O2—C7	177.1 (4)
C11—C12—C13—C14	0.0 (10)	C13—C14—O3—C15	2.5 (8)
C12—C13—C14—O3	179.5 (6)	C9—C14—O3—C15	−177.8 (5)
C12—C13—C14—C9	−0.3 (9)	C16—C15—O3—C14	−172.6 (5)
C10—C9—C14—O3	−178.8 (5)	C18—C17—O4—C16	−6.4 (10)
C8—C9—C14—O3	1.8 (7)	C22—C17—O4—C16	173.5 (5)
C10—C9—C14—C13	1.0 (9)	C15—C16—O4—C17	105.9 (7)
C8—C9—C14—C13	−178.4 (5)		

### Hydrogen-bond geometry (Å, °)

**Table:**

*D*—H···*A*	*D*—H	H···*A*	*D*···*A*	*D*—H···*A*
C1—H1···O2	0.93	2.42	2.753 (9)	101
C10—H10···O2	0.93	2.35	2.710 (8)	102
C23—H23···O4	0.98	2.19	2.700 (8)	111
C5—H5···Br1^i^	0.93	3.03	3.529 (7)	116

Symmetry codes: (i) −*x*+1, *y*+1/2, −*z*+1/2.
